# Biological and Structural Analyses of New Potent Allosteric Inhibitors of HIV-1 Integrase

**DOI:** 10.1128/aac.00462-23

**Published:** 2023-06-13

**Authors:** Damien Bonnard, Erwann Le Rouzic, Matthew R. Singer, Zhe Yu, Frédéric Le Strat, Claire Batisse, Julien Batisse, Céline Amadori, Sophie Chasset, Valerie E. Pye, Stéphane Emiliani, Benoit Ledoussal, Marc Ruff, François Moreau, Peter Cherepanov, Richard Benarous

**Affiliations:** a Biodim, Romainville, France; b Chromatin Structure and Mobile DNA Laboratory, Francis Crick Institute, London, United Kingdom; c IGBMC, INSERM, CNRS, Université de Strasbourg, Illkirch, France; d Université Paris Cité, Institut Cochin, INSERM, CNRS, Paris, France; e Department of Infectious Disease, St. Mary's Campus, Imperial College London, London, United Kingdom

**Keywords:** HIV-1, integrase, LEDGF, antiretrovirals, drug discovery, allosteric inhibitor, ALLINI, protein-protein interaction inhibitor, integrase inhibitor, molecular glue, cocrystallization.

## Abstract

HIV-1 integrase-LEDGF allosteric inhibitors (INLAIs) share the binding site on the viral protein with the host factor LEDGF/p75. These small molecules act as molecular glues promoting hyper-multimerization of HIV-1 IN protein to severely perturb maturation of viral particles. Herein, we describe a new series of INLAIs based on a benzene scaffold that display antiviral activity in the single digit nanomolar range. Akin to other compounds of this class, the INLAIs predominantly inhibit the late stages of HIV-1 replication. A series of high-resolution crystal structures revealed how these small molecules engage the catalytic core and the C-terminal domains of HIV-1 IN. No antagonism was observed between our lead INLAI compound BDM-2 and a panel of 16 clinical antiretrovirals. Moreover, we show that compounds retained high antiviral activity against HIV-1 variants resistant to IN strand transfer inhibitors and other classes of antiretroviral drugs. The virologic profile of BDM-2 and the recently completed single ascending dose phase I trial (ClinicalTrials.gov identifier: NCT03634085) warrant further clinical investigation for use in combination with other antiretroviral drugs. Moreover, our results suggest routes for further improvement of this emerging drug class.

## INTRODUCTION

Integrase (IN) is the essential retroviral enzyme responsible for insertion of the reverse transcribed viral genome into a host cell chromosome (1). HIV-1 IN strand transfer inhibitors (INSTIs) raltegravir (RAL) and elvitegravir (EVG) were introduced in the therapeutic arsenal against HIV-1 in 2007 and 2012, respectively (2). The second-generation INSTI dolutegravir (DTG) became available in 2013, followed by bictegravir (BIC), and, more recently, cabotegravir (3–5). The INSTIs performed remarkably well in clinic, with the second-generation INSTIs displaying a considerably improved profile of resistance. However, viruses resistant to the second-generation compounds have been reported, particularly in patients with a long history of multiple anti-HIV-1 treatments that suffer multiresistance to antiretrovirals (6, 7). It is thus of paramount importance to continue developing compounds with alternative modes of action that retain activity against viruses resistant to currently available drugs. Particularly promising among the emerging antiretrovirals are allosteric inhibitors of HIV-1 IN (8). Small molecules belonging to this novel class of HIV-1 inhibitors were initially reported in a Boehringer Ingelheim patent (9) and then by Zeger Debyser’s laboratory (10). Later, several other pharmaceutical companies and academic groups reported many more lead compounds belonging to this class (11–19). Consequently, the molecules became known by alternative names: LEDGINs (10, 19), allosteric integrase inhibitors (ALLINIs) (11, 18), noncatalytic integrase inhibitors (NCINIs) (9, 13, 15, 17), multimerization integrase inhibitors (MINIs) (11, 14), and IN-LEDGF allosteric inhibitors (INLAIs) (12, 16). Herein, these small molecules are collectively referred to as INLAIs. Two members of this class, namely, BDM-2, described in this work, and STP0404 (Pirmitegravir) have completed phase 1 clinical trials (20–22). INLAIs bind to HIV-1 IN and compete with LEDGF/p75, the host factor that directs lentiviral integration toward actively expressed genes (23–27). Unusually, INLAIs affect HIV-1 replication via three separate mechanisms. As competitors of LEDGF/p75, the small molecules inhibit HIV-1 integration and perturb the preference of the virus to integrate into active transcription units and consequently reduce subsequent proviral expression (28–30). Finally, INLAIs promote aberrant hyper-multimerization of HIV-1 IN resulting in production of defective progeny virions that harbor genomic RNA mislocalized outside the mature capsid core. Such virions are unable to complete reverse transcription upon entry into new cells (23, 31–34). Although HIV-1 IN was shown to bind viral genomic RNA (35, 36), the precise mechanism of IN-dependent RNA incorporation into viral cores remains to be elucidated. Because INLAIs are far more potent at inhibiting the production of infectious HIV-1 particles than the integration step (12, 13, 23, 31), these small molecules can be broadly classified as HIV-1 maturation inhibitors.

The primary INLAI-binding site on HIV-1 IN is a pocket at the core catalytic domain (CCD) dimerization interface. To engage it, the compounds use a conserved warhead module comprising three chemical functions: a carboxyl, a *tert*-butoxy, and a bulky aromatic chemical group attached to an aromatic scaffold (1, 37). In their mode of binding to HIV-1 IN, INLAIs mimic the LEDGF/p75 integrase binding domain (IBD), which engages the same pocket on the CCD dimer interface (38). Recent X-ray crystallography and biochemical studies revealed that INLAIs act as molecular glues, promoting formation of a pathological interface involving the HIV-1 CCD dimer and a C-terminal domain (CTD) (39–42). While HIV-1 IN forms stable tetramers (24, 43, 44) under physiological conditions, the formation of the additional, drug-induced IN-IN interface leads to uncontrolled multimerization of the viral protein. Recently, the inhibitor-mediated CCD-CTD interface was visualized in high-resolution cocrystal structures of a two-domain HIV-1 IN construct with INLAIs BI-D and STP0404 (45). These structures revealed that while occupying their principal binding pocket on the HIV-1 IN CCD dimerization interface, the compounds protrude sufficiently to recruit several highly conserved residues of the CTD.

Herein, we describe a new series of highly potent INLAIs that block HIV-1 replication at low nanomolar concentrations, expanding the chemical diversity of this promising class of antivirals. We determined cocrystal structures of these compounds with HIV-1 IN, which explain their high potency as antiretroviral agents. We characterized INLAI-resistant HIV-1 strains and demonstrate a lack of antagonism between these compounds and several anti-HIV drugs used in clinic.

## RESULTS

### Novel INLAIs based on a benzene scaffold.

**FIG 1:**
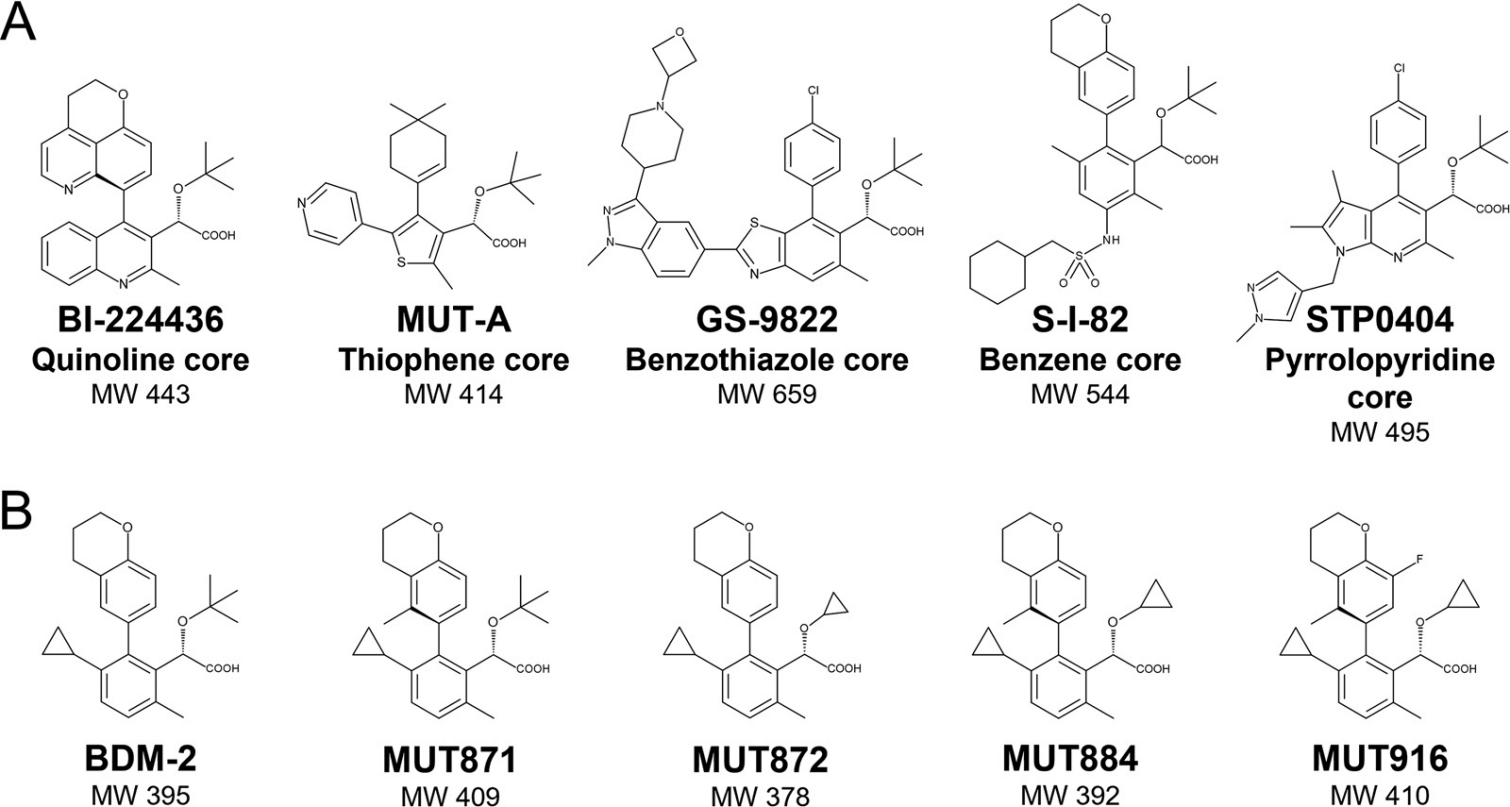
Chemical structures of INLAIs. (A) Previously reported INLAIs are built upon different scaffolds, as indicated. All these compounds share the same active group characterized by a carboxylic acid linked to a *tert*-butoxy moiety. (B) Lead compound BDM-2 series. These molecules have a benzene scaffold and contain either a *tert*-butoxy (BDM-2, MUT871), or a cyclopropyloxy group (MUT872, MUT884 and MUT916). MW, molecular weight. These molecules were developed by Biodim and are today property of Mutabilis (Romainville, France).

A range of scaffolds, including quinoline for BI-224436 (15), thiophene for MUT-A (16), benzene for Shionogi S-I-82 (46), benzothiazole for GS-9822 (47), and pyrrolopyridine for STP0404 (21), have been used for the development of INLAIs (Fig. 1A). Importantly, all described potent INLAIs share the common chemical module comprising a carboxylic and a *tert*-butoxy moiety attached to a bulky hydrophobic side chain via an aromatic scaffold, which are collectively responsible for binding to the pocket on HIV-1 IN CCD dimer interface (1). Biodim (Romainville, France) has developed novel potent INLAIs, including BDM-2 (the lead compound of the series), MUT871, MUT872, MUT884, and MUT916, all of which are investigated in this paper (Fig. 1B). The BDM-2 series of INLAIs is based on a benzene scaffold and the compounds contain either the carboxyl and *tert*-butoxy groups (BDM-2 and MUT871) or a derivative of this motif, with the *tert*-butoxy side chain replaced by a cyclopropyloxy group (MUT872, MUT884, and MUT916). Compound MUT871 differs from BDM-2 by a methyl substituent on the chromane group.

### The BDM-2 series of INLAIs inhibit HIV-1 IN-LEDGF/p75 interaction and promote IN hyper-multimerization.

The INLAIs were tested for the ability to inhibit the HIV-1 IN-LEDGF/p75 interaction in a homogeneous time-resolved fluorescence (HTRF) assay. Our assays used either full-length IN and LEDGF/p75 proteins or their respective minimally sufficient interaction domains: the CCD of HIV-1 IN and the IBD of LEDGF/p75 (Table 1). The most potent inhibitor of the IN-LEDGF/p75 interaction of all INLAIs tested here was MUT871 with 50% inhibitory concentration (IC_50_) of 14 nM, compared to 90 and 820 nM for previously described inhibitors of this class BI-224436 and S-I-82, respectively. The remaining compounds from the BDM-2 series, MUT872, MUT884, MUT916, and BDM-2, were also very potent at disrupting the IN-LEDGF/p75 complex formation, yielding IC_50_ values in the range between 17 nM for MUT872 and 62 nM for MUT884 (Table 1). The interaction between isolated HIV-1 IN CCD and LEDGF/p75 IBD was likewise susceptible to the compounds, although BDM-2, MUT871, and MUT872 appeared to be more active at inhibiting full-length IN-LEDGF/p75 complex formation (Table 1).

**TABLE 1:** Biochemical activities of INLAIs in HIV-1 IN-LEDGF/p75 interaction and HIV-1 IN homo-multimerization assays^*a*^

Compound	IN-LEDGF/p75 interaction	CCD-IBD interaction	IN multimerization^*b*^	
	IC_50_ (μM)	IC_50_ (μM)	AC50 (μM)	Plateau (%)
BI-224436	0.090 ± 0.019	0.073 ± 0.013	0.034 ± 0.011	670 ± 210%
S-I-82	0.82 ± 0.38	0.43 ± 0.13	0.047 ± 0.031	500 ± 43%
BDM-2	0.047 ± 0.014	0.15 ± 0.03	0.020 ± 0.011	560 ± 150%
MUT871	0.014 ± 0.005	0.032 ± 0.014	0.031 ± 0.020	440 ± 53%
MUT872	0.17 ± 0.02	0.42 ± 0.07	0.043 ± 0.011	440 ± 48%
MUT884	0.062 ± 0.009	0.082 ± 0.021	0.022 ± 0.004	400 ± 71%
MUT916	0.046 ± 0.016	0.019 ± 0.008	0.022 ± 0.002	370 ± 60%

a Data are mean ± SD of *n* > 3 independent experiments.

b Promotion of IN multimerization, with compound concentration required for 50% maximum activation of multimerization (AC_50_) and maximum multimerization reached versus baseline (Plateau).

A key property of INLAIs is the ability to promote HIV-1 IN homo-multimerization (39–42). Concordantly, BDM-2 and its analog compounds enhanced IN aggregation, with 50% of maximum activation (AC_50_) obtained in the concentration range like that required for the inhibition of IN-LEDGF/p75 interaction. The highest activity, 20 nM, was observed for BDM-2, compared to 43 nM for MUT872, 34 nM for BI-224436, and 47 nM for S-I-82 (Table 1). For all compounds studied here, except MUT871, the AC_50_ of IN multimerization was lower than the respective IC_50_ of the IN-LEDGF/p75 interaction. MUT871, for which the AC_50_ is slightly higher than that of IC_50_, is our most potent inhibitor of IN-LEDGF/p75 interaction. This result confirms that INLAIs tend to be more potent as IN-IN molecular glues than as inhibitors of IN-LEDGF interaction (12, 16). The maximal signal (plateau) in our IN aggregation assay corresponds to the highest HTRF signal increase under saturating INLAI concentration, relative to baseline level. Thus, MUT916, MUT884, MUT871, and MUT872 increased HIV-1 IN aggregation 3.7 to 4.4-fold, while BDM-2, BI-224436, and S-I-82 did so 5 to 6-fold (Table 1).

### The BDM-2 series compounds are potent inhibitors of HIV-1 replication.

Antiretroviral activity of BDM-2 and its analogs was first evaluated in lymphoblastoid cell line MT4 and in primary human T lymphocytes using NL4-3 and HXB2 laboratory HIV-1 isolates. The inhibitory potencies were compared to that of the INSTIs RAL and DTG, and to the previously described INLAIs BI-224436 and S-I-82 (Table 2). BDM-2 and MUT871 were the most potent INLAIs of this series, with 50% effective concentrations (EC_50_s) 8.7 nM and 3.1 nM against NL4-3, and 4.5 nM and 1.4 nM against HXB2, respectively. The remaining compounds of the BDM-2 series also displayed high potency, with EC_50_s in the range of 6.3 to 18 nM and 15 to 45 nM against HXB2 and NL4-3, respectively (Table 2). In the absence of human serum, BDM-2 and MUT871 were more potent than RAL, while the antiviral activity of MUT871 approached that of DTG (EC_50_ of 1.9 nM and 2.7 nM against HXB2 and NL4-3, respectively). All BDM-2 series compounds displayed higher antiviral activity than the previously reported INLAI BI-224436, while BDM-2 matched S-I-82 in potency. Moreover, the compounds had a favorable cytotoxicity profile with 50% cytotoxic concentrations (CC_50_s) in the range of 46 to 139 μM and consequently very high selectivity indexes (Table 2).

**TABLE 2:** Antiviral activities, cytotoxicity, and selectivity indices of BDM-2 series determined on MT4 cells infected with HXB2 or NL4-3 HIV-1 strains^*a*^

Compound	Antiviral activity in multiple-round infection assays in MT4 cells						
	EC_50_ (nM) HXB2	EC_50_ (nM) NL4-3	EC_90_ (nM) NL4-3	PA-EC_90_ (nM) NL4-3	PA-EC_90_ (ng/mL) NL4-3	Cytotoxicity CC_50_ (μM)	Selectivity index CC_50_/EC_50_ NL4-3
RAL	8.7 ± 6.0	14 ± 9	28 ± 14	105 ± 38	46 ± 17	438 ± 29	31,285
DTG	1.9 ± 1.0	2.7 ± 1.3	6.3 ± 4.4	159 ± 144	67 ± 60	51 ± 28	18,888
BI-224436	23 ± 8.4	51 ± 17	77 ± 34	273 ± 103	121 ± 46	>200	>3,921
S-I-82	4.1 ± 0.7	12 ± 3	21 ± 7.2	NT	NT	28 ± 3	2,333
BDM-2	4.5 ± 1.2	8.7 ± 2.8	13 ± 5.1	127 ± 53	50 ± 21	69 ± 8	7,931
MUT871	1.4 ± 0.3	3.1 ± 1.0	4.6 ± 1.6	98 ± 31	40 ± 13	46 ± 11	14,838
MUT872	18 ± 1	45 ± 12	61 ± 19	999 ± 373	378 ± 141	139 ± 29	3,088
MUT884	6.3 ± 1.1	15 ± 5	23 ± 13	439 ± 124	172 ± 49	78 ± 12	5,200
MUT916	8.8 ± 0.8	20 ± 9	30 ± 15	865 ± 418	355 ± 172	78 ± 6	3,900

a Evaluation of antiviral activity by EC_50_ and protein-adjusted EC_90_ (PA-EC_90_), determination of cytotoxicity (CC_50_) on MT4 cells, and selectivity index (defined as CC_50_/EC_50_ ratio) values. Two INSTIs (RAL, DTG), and two previously reported INLAIs (BI-224436, S-I-82) were used as references. Data are mean ± SD of *n* > 5 independent experiments; NT, not tested.

EC_90_ values for the compounds, inferred from the respective dose response curves, are given in Table 2. We determined the effect of human serum on the antiviral potency by extrapolation of the EC_90_ at 100% human serum. This value corresponding to the protein-adjusted EC_90_ (PA-EC_90_), was estimated from the experimental antiviral activities in MT4/NL4-3 infection assays in the presence of 10 to 50% human serum. Thus, PA-EC_90_ values of BDM-2 and MUT871 were estimated as 127 nM (corresponding to 50 ng/mL) and 98 nM (40 ng/mL), respectively (Table 2). PA-EC_90_ is an important parameter used to determine the clinical inhibitory quotient (IQ) to guide the selection of human dose of drug candidates. BDM-2 PA-EC_90_ values are comparable to that of DTG (3) and are more favorable than that of the previously reported INLAI BI-224436 (121 ng/mL). BDM-2, MUT871, and MUT884 compounds potently inhibited replication of lab-adapted and clinical HIV-1 isolates in primary CD4^+^ T lymphocyte cultures (Table 3). Of note, the antiretroviral potency of BDM-2 was similar to that of S-I-82 and exceeded activity of BI-224436 or RAL, while the potency of MUT871 was the highest of all INLAIs and on pair with DTG.

**TABLE 3:** Antiretroviral activity of BDM-2 and two representative analogs on cultures of primary T CD4^+^ lymphocytes infected with lab-adapted and clinical HIV-1 isolates

Compound	Infection assays in primary T CD4^+^ lymphocytes: EC_50_ (nM)^*a*^					
	NL4-3	HXB2	KER2008	33913N	NP1538	NP1525
	(B)	(B)	(A)	(B)	(B)	(CRF01_AE)
RAL	4.9 ± 2.3	2.6 ± 1.4	6.7 ± 4.0	2.1 ± 0.1	1.7 ± 1.9	4.0 ± 1.7
DTG	0.53 ± 0.16	0.17 ± 0.01	0.40 ± 0.21	0.35 ± 0.42	0.29 ± 0.13	0.21 ± 0.09
BI-224436	100 ± 80	14 ± 7	37 ± 12	20 ± 9	66 ± 42	61 ± 35
S-I-82	9.9 ± 1.1	5.2 ± 3.9	11 ± 2	4.4 ± 1.9	8.6 ± 0.5	10 ± 4
BDM-2	8.3 ± 4.3	3.4 ± 1.0	8.1 ± 1.3	4.2 ± 1.6	13 ± 6	11 ± 5
MUT871	2.8 ± 0.5	1.8 ± 0.3	6.4 ± 1.3	1.5 ± 0.6	4.0 ± 2.3	3.5 ± 0.6
MUT884	15 ± 4	10 ± 6	19 ± 3	5.8 ± 3.2	41 ± 2	12 ± 3

a EC_50_ values are reported for multiple-round infection assays for each HIV-1 strain (clade indicated in parenthesis). Two INSTIs (RAL, DTG) and two INLAIs (BI-224436, S-I-82) were used as references. Data are mean ± SD of *n* > 5 independent experiments.

### Dual antiretroviral activities of INLAIs.

To dissect the mechanism of action of the BDM-2 series compounds, we compared their antiviral activities in single- and multiple-round HIV-1 NL4-3 infection assays. Single-round infection assays utilize pseudotyped, replication-defective viral particles (produced in the absence of inhibitors) and report on the ability of a compound to block the early steps of the HIV-1 replication cycle (from entry into host cells to provirus expression). In contrast, multiple-round infection assays use a replication competent virus to assess antiviral activity over multiple complete infection cycles. As expected, INSTIs were highly potent at inhibiting HIV-1 both in single- and multiple-round assays, with similar EC_50_ values (Tables 2 and 4). By sharp contrast, all INLAIs tested here, including BDM-2 and MUT871, possessed much greater antiviral activity in the multiple-round assays. Thus, the INLAI EC_50_ values determined in single- and multiple-round infection assays were in the micromolar (0.63 to 9.2 μM, Table 2) and nanomolar range (3.1 to 51 nM, Table 4), respectively. These results are in line with previous observations that INLAIs predominantly inhibit the late stages of the HIV-1 replication cycle (12, 13, 23, 31).

**TABLE 4:** Antiretroviral activities of the BDM-2 series in single-round compared to multiple-round infectivity assays with NL4-3 HIV-1 isolate in MT4 cells^*a*^

Compound	EC_50_ in single-round infection (μM)	EC_50_ ratio (single/multiple-round infection)^*b*^
RAL	0.0035 ± 0.0010	0.25
DTG	0.00044 ± 0.00013	0.16
BI-224436	1.4 ± 0.3	27
S-I-82	9.2 ± 1.1	767
BDM-2	1.4 ± 0.3	161
MUT871	0.63 ± 0.01	203
MUT872	8.8 ± 0.3	196
MUT884	3.9 ± 0.8	260
MUT916	2.1 ± 0.2	105

a EC_50_ values were determined in the single-round infectivity assay using a single-cycle NL4-3 HIV-1 clone. Two INSTIs (RAL, DTG) and two INLAIs (BI-224436, S-I-82) were used as references. Data are mean ± SD of *n* > 5 independent experiments.

b Ratios of EC_50_ obtained in the single- and multiple-round infection assays. The corresponding EC_50_ from multiple-round infection assays are given in Table 2.

Intriguingly, the ratios of EC_50_ values determined in the single- and multiple-round assays varied considerably between INLAIs, ranging from ~30 for BI-224436 to ~800 for S-I-82 (Table 4). Specifically, for BDM-2 and its analogs, the EC_50_ ratios were in the range of 100 to 200 (Table 4). These variations may reflect differences in the dual biochemical activities of these compounds, namely, in their ability to inhibit IN–LEDGF/p75 interaction versus their action as molecular glues promoting IN aggregation. Indeed, there is a striking correlation between the IC_50_ value of an INLAI in its ability to disrupt the IN-LEDGF/p75 complex (Table 1) and its antiviral potency in single-round infection assay (Table 4). The INLAI compound with the best IC_50_ in disrupting IN-LEDGF/p75 interaction, MUT871, is also the most potent in the single-round assay. The weakest compound to inhibit IN-LEDGF/p75, S-I-82, is also the weakest in antiviral activity in single-round infection assays.

### Antiretroviral activity of the BDM-2 series on polymorphic recombinant and primary HIV-1 isolates.

Located on the HIV-1 IN CCD dimerization interface, the principal INLAI binding pocket overlaps with a hot spot of amino acid sequence polymorphism involving IN residues 124 and 125 (16). To test if the new INLAIs maintained antiviral activity against major polymorphisms, we introduced all major combinations of residues found in circulating HIV-1 strains at positions 124 and 125 into NL4-3 HIV-1 molecular clone. In total, we evaluated 15 such combinations that collectively covered 98% of all HIV-1 clade polymorphisms involving these positions (48). To gauge effects on the antiviral activity, we calculated the EC_50_ fold change (FC) relative to wild type NL4-3 HIV-1 strain that features Thr124 and Thr125 (*TT*; specific polymorphisms are referred to by a single letter amino acid code). BDM-2 and its analogs maintained their potency against almost all 15 polymorphisms tested, with the corresponding FC values of 1 (Table 5). Particularly, BDM-2 displayed an FC of 1 for all polymorphisms except *AV*, which increased the FC to 2. MUT871 likewise performed well, with only the *GT*, *GA*, and *NA* polymorphisms increasing the FC to 2, 3, and 2, respectively. For MUT916, the only polymorphism with FC >1 was *NA*, with an FC of 4. The FC of 3-fold or less can be considered modest, demonstrating that this BDM-2 series is associated with broad polymorph coverage. By sharp contrast, S-I-82 lost most of its antiviral activity against HIV-1 strains with an Asn residue at position 124, displaying an FC of 10 for *NA*, and an FC of 5 for *NT* and *NV* viruses.

**TABLE 5:** Antiviral activities of the BDM-2 series against recombinant viruses constructed in NL4-3 background with amino acid polymorphisms at IN positions 124 and 125^*a*^

Compound	Fold change in EC_50_ relative to parental NL4-3 in multiple-round infection of MT4 cells													
	AA	AM	AT	AV	GT	GA	NA	NT	NV	SA	ST	TA	TM	TV
DTG	1	2	1	2	2	2	1	1	2	1	2	1	2	2
BI-224436	1	0.4	1	1	2	1	1	1	1	1	1	1	0.4	1
S-I-82	1	1	1	2	3	3	10	5	5	4	2	2	1	1
BDM-2	1	0.4	1	2	1	1	1	1	1	1	1	1	0.3	1
MUT871	1	0.4	1	1	2	3	2	1	1	1	1	1	0.3	1
MUT872	1	0.3	1	0.5	2	2	1	1	1	1	1	1	0.2	1
MUT884	0.4	0.2	1	0.5	2	1	2	1	1	1	1	1	0.2	1
MUT916	1	0.2	0.5	0.4	1	1	4	1	0.4	1	1	1	0.2	0.4

a The polymorphisms at IN amino acid positions 124/125 of the recombinant NL4-3 polymorphic viruses are indicated with two-letter codes (AA, AM, AT, AV, GT, GA, NA, NT, NV, SA, TA, TM, or TV). The levels of retained antiviral activity are reported by EC_50_ fold change compared to the wild type NL4-3 HIV-1 clone. Fold change values >10 highlighted in dark gray, and those in the range of 5 to 10 in light gray. One INSTI (DTG) and two INLAIs (BI-224436, S-I-82) were used as references. Data are mean ± SD of *n* > 5 independent experiments.

Next, we evaluated the potency of the BDM-2 series of INLAIs against several primary HIV-1 isolates with polymorphisms at IN positions 124 and 125. As shown in Table 6, BDM-2 compounds maintained excellent antiviral activity against all 14 primary isolates tested, with corresponding FCs of 1 to 3, except for primary isolate vGA, which was inhibited by BDM-2 and analogs with FCs of 4 to 5. In addition, MUT916 lost substantial activity against vNA_2. In agreement with the results obtained with recombinant NL4-3 variants, S-I-82 displayed more severe losses against polymorphic primary HIV-1 isolates, particularly against primary isolates featuring *NA* or *NV* polymorphisms with FCs of 210, 23, 23, 34, respectively, and a FC of 12 for the primary isolate vSA_1.

**TABLE 6:** Antiviral activities of the BDM-2 series against primary HIV-1 isolates with amino acid polymorphisms at IN positions 124 and 125^*a*^

Compound	Fold change in EC_50_ relative to parental NL4-3 in multiple-round infection of MT4 cells																
	AA						GA		NA				NV	SA		TA	
	KER2008 (A)	TZA125 (C)	E0836M4 (D)	NP1525 (CRF01)	NP1695 (CRF02)	CAM2BBY (CRF02)	KSM4030 (A)	vGA (C)	NP1538 (B)	99ET14 (C)	vNA_1 (B)	vNA_2 (CRF02)	vNV (B)	vSA_1 (B)	vSA_2 (CRF02)	vTA_1 (B)	vTA_2 (CRF02)
DTG	1	2	2	2	2	1	2	2	1	2	2	2	1	1	2	1	1
BI-224436	1	0.5	1	1	2	2	1	2	1	1	2	2	1	3	2	2	2
S-I-82	1	1	1	2	1	2	6	4	1	210	23	23	34	12	4	3	3
BDM-2	1	0.4	1	1	2	2	2	4	2	1	2	3	2	3	3	2	1
MUT871	1	1	2	1	3	2	2	5	2	2	3	3	3	4	3	2	2
MUT872	1	0.4	1	1	1	1	2	4	2	1	1	2	1	2	2	2	2
MUT884	0.5	0.3	1	1	2	1	1	5	3	1	3	4	2	2	2	1	2
MUT916	1	0.4	1	1	2	1	1	5	2	2	4	10	1	3	4	2	2

a The polymorphisms at IN amino acid positions 124/125 of the primary HIV-1 isolates are indicated with two-letter codes (AA, GA, NA, NV, SA, or TA), the clade of each of the HIV-1 primary isolates is given in parenthesis. The levels of retained antiviral activity are reported by EC_50_ fold change compared to the wild type NL4-3 HIV-1 clone. Fold change values >10 highlighted in dark gray, and those in the range of 5 to 10 in light gray. One INSTI (DTG) and two INLAIs (BI-224436, S-I-82) were used as references. Data are mean ± SD of *n* > 5 independent experiments.

### BDM-2 is fully active on viruses resistant to all classes of current clinical antiretrovirals.

Next, we wished to confirm that BDM-2, as a representative lead compound of this new series of INLAIs, retained activity against HIV-1 isolates that have acquired resistance to the drugs currently used in clinic. To this end, we used both recombinant viruses constructed by introducing described drug resistance mutations into NL4-3 HIV-1 clone as well as drug-resistant clinical HIV-1 isolates. As expected, BDM-2 maintained high inhibitory activity against HIV-1 strains resistant to the orthogonal classes of antiretrovirals, including INSTIs, nucleoside and nonnucleoside reverse transcriptase inhibitors (NRTIs and NNRTIs), as well as protease inhibitors (PIs), with FC values of 1 for all drug-resistant strains, except an FC of 2 for NRTI-resistant strain NLAY351717 (Table 7).

**TABLE 7:** Activities of the BDM-2 series against HIV-1 strains resistant to drugs currently used in clinic^*a*^

Drug class	Compound	Fold change in EC_50_ in multiple-round infection assays relative to NL4-3																	
		INSTIs									NRTI/NNRTIs			PIs					
		E92Q	Y143R	N155H	T97A/N155H (pat.)	G140S	Q148H	G140S/Q148H	G140S/Q148H (pat.)	E92Q/N155H (pat.)	NLAY351717	NLAY351753	K103N/Y181C	ARP-4595	ARP-4596	ARP-11801	ARP-11803	ARP-11807	ARP-11808
INLAI	BDM-2	1	1	1	1	1	1	1	1	1	2	1	1	1	0.5	1	1	1	1
	MUT871	1	1	1	NT	1	1	1	NT	NT	1	1	1	1	1	1	1	1	1
	MUT872	1	0.5	1	NT	1	1	1	NT	NT	1	1	1	1	1	2	1	1	2
	MUT884	1	0.5	1	NT	1	1	1	NT	NT	1	1	1	1	1	3	2	1	2
	MUT916	1	0.4	1	NT	1	1	1	NT	NT	1	2	1	2	1	2	2	2	2
	BI-224436	1	1	1	1	1	1	1	1	1	1	1	1	0.4	0.5	1	1	1	1
	S-I-82	NT	NT	NT	NT	NT	NT	NT	NT	NT	NT	NT	NT	1	0.5	1	1	1	1
INSTI	RAL	3	14	6	140	1	9	190	440	31	1	1	1	1	1	1	1	2	2
	EVG	24	2	12	440	2	3	2500	2000	200	1	1	1	1	1	1	1	2	2
	DTG	NT	0.4	0.3	3	0.6	0.3	2	3	1	0.4	1	0.3	1	0.5	1	1	2	1
NRTI	AZT	1	1	1	4	1	1	1	1	1	150	210	0.1	2	1	1	2	2	5
NNRTI	EFV	1	1	1	2	1	1	1	1	1	2	39	51	1	1	0.5	1	2	1
	RPV	NT	NT	NT	1	1	1	1	1	1	1	0.4	3	1	1	0.4	1	1	1
PI	IDV	1	1	1	NT	NT	NT	1	NT	NT	1	1	1	5	8	92	260	36	59
	DRV	NT	NT	NT	NT	NT	NT	1	NT	NT	1	1	1	1	1	31	24	12	240

a Activities of INLAIs and clinical antiretrovirals against HIV-1 strains resistant to INSTIs, nucleoside reverse transcriptase inhibitors (NRTIs), nonnucleoside reverse transcriptase inhibitors (NNRTIs), or protease inhibitors (Pis). HIV-1 strains are indicated by their respective resistance mutations (in the NL4-3 background) or by specific clone names. The NRTI and NRTI/NNRTI inhibitor-resistant clones NLAY351717 and NLAY351753 contained RT coding regions from GenBank sequence identifiers AY351717 and AY351753, respectively. The representative PI-resistant viruses ARP-4595 (harboring mutations M46I/L63P/V82T/I84V in the protease coding region), ARP-4596 (L10R/M46I/L63P/V82T/I84V), ARP-11801 (L10F/G16A/L33F/K43T/L63P/A71V/V82A), ARP-11803 (L33F/E34T/K43T/G48V/I54S/L63P/A71V/V82A), ARP-11807 (L33F/I47A/L63P/A71V/V82A), and ARP-11808 (L10F/T12P/L19P/K20T/L33F/E35G/L63P/K70T/A71V/G73S/P79A) were obtained from the NIH AIDS Reagent Program. Pat. Indicates primary patient-derived strain. Antiviral activities reported as EC_50_ fold change compared to wild type NL4-3 HIV-1 strain. Fold-change values >5 and >50 highlighted in light and dark gray, respectively. The reference compounds included examples of each drug class as well as two other INLAIs (BI-224436, S-I-82). EFV, efavirenz; RPV, rilpivirine; IDV, indinavir; DRV, darunavir. NT, not tested.

### Selection of HIV-1 resistance to BDM-2.

**FIG 2:**
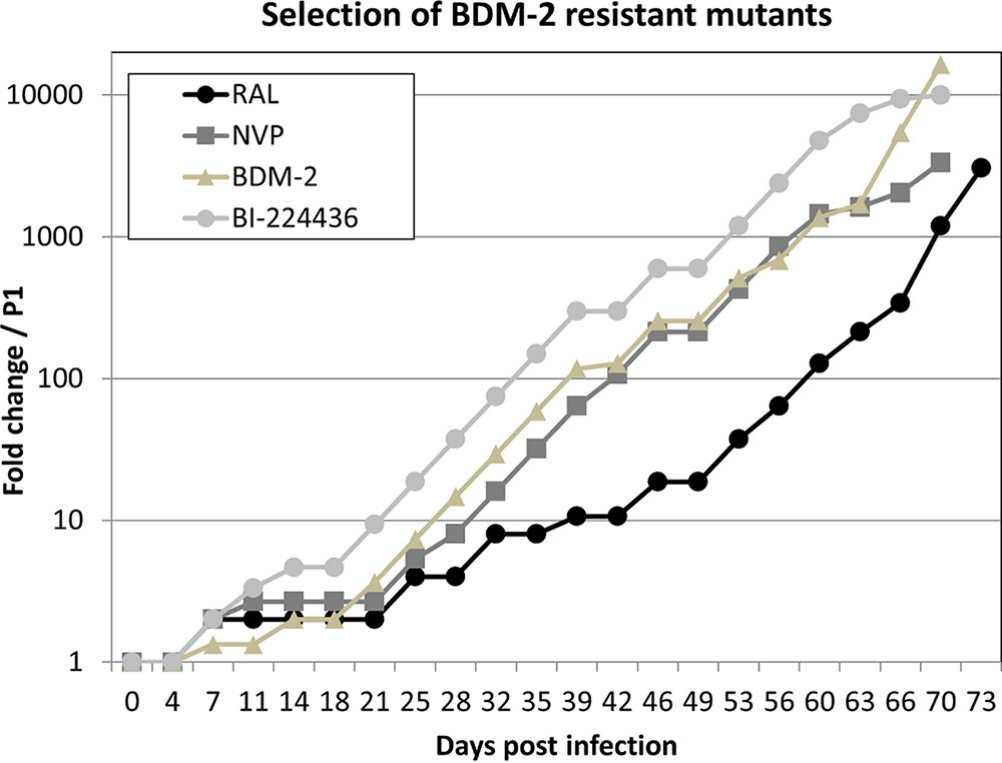
*In vitro* selection of HIV-1 resistance to BDM-2, BI-224436, raltegravir (RAL), and nevirapine (NVP). Replication of the NL4-3 HIV-1 clone in a culture of MT4 cells was monitored under escalating drug pressure. The selection process was initiated at the drug concentration corresponding to the EC_50_ value for each drug. Virus replication was monitored during 73 days by fold change in EC_50_ of the inhibitors used in these experiments.

To evaluate the genetic barrier to BDM-2 resistance, we propagated the NL4-3 HIV-1 strain under escalating compound levels. In parallel, we followed selection of resistance to three reference compounds: a clinical INSTI (RAL), a clinical NNRTI (nevirapine [NVP]), and an unrelated INLAI (BI-224436). The progress of virus replication in the presence of stepwise increasing concentrations of inhibitors was monitored over time (Fig. 2). The emergence of phenotypic resistance to BDM-2 and BI-224436 was similar to that of NVP and occurred slightly faster than to RAL. These results confirmed that INLAIs display relatively low genetic barrier to resistance, which is like that of NNRTIs and likely lower than INSTIs.

To monitor emergence of genotypic resistance, we sequenced entire IN-coding regions within viral genome populations at various time points during the selection process (Table 8). The occurrence frequency of individual missense mutations was estimated as a percentage of the total number of missense mutations observed within the viral culture at each time point. Early changes detected at day 18 were amino acid substitutions close to the INLAI binding site in the IN-CCD: A128T, the most frequent one found in 50% of IN sequences, and Y99H found in 36% of IN sequences. Interestingly, a low level (7%) of N222K mutation in the CTD domain was also detected at that time point. At the intermediate time point, 42 days postinfection, the landscape of INLAI resistance mutations did not change drastically. The most detrimental mutations such as T174I, and to a lesser degree A129T, occurred late during the selection process, between days 66 and 73, while previously detected mutations A128T and N222K were still present in IN sequences. Identical mutations with similar kinetics were also detected during the selection process with BI-224436 (Fig. 2 and Table 8).

**TABLE 8:** Kinetics of occurrence of INLAI-resistant mutants^*a*^

Compound	Mutation	Kinetic (avg %)		
		Early	Intermediate	Final
BDM-2	Y99H	36	31	23
	T125A	0	0	3
	A128T	57	50	30
	A129T	0	0	10
	E170G	0	0	3
	H171Q	0	0	3
	L172I/F	0	0	3
	T174I	0	0	10
	N222K	7	19	13
BI-224436	Y99H	0	11	13
	A128T	40	33	38
	E170G	20	11	0
	H171Q	20	11	13
	T174I	0	22	25
	N222K	20	11	13

a Frequency of occurrence of missense mutations detected within IN-coding region during selection with BDM-2 and BI-224436 at three different time points, early (18 days postinfection [dpi]), intermediate (42 dpi) and final (66 to 73 dpi). The frequency of each identified missense mutation was estimated as a percentage of the total number of missense mutations detected in IN sequences from each sample. The most and second-most prevalent mutations are highlighted in dark and light gray, respectively.

To confirm that the observed genetic changes are responsible for the observed phenotypic resistance, we introduced major single point mutations identified during selection into NL4-3 HIV-1 clone. The resulting mutants were tested for sensitivity to the compound (Table 9). The most detrimental mutation for all INLAIs, including BDM-2, was T174I with FC values of 253, 706, and 15 for BDM-2, BI-224436, and S-I-82, respectively. The remaining single point mutations detected during the selection process, namely, Y99H, A128T, H171Q, and N222K conferred much weaker resistance with the corresponding FC values in the range of 2 to 4. All INLAIs investigated shared a similar resistance profile to that of compounds of the BDM-2 series. These results are consistent with the resistance profile for previously reported INLAIs. As expected, EVG as an INSTI representative of current drugs used in clinic, maintained full activity against all INLAI-resistant mutants.

**TABLE 9:** Antiviral activity of the BDM-2 series against viruses harboring single point mutations selected by INLAIs^*a*^

Compound	EC_50_ (nM)	EC_50_ fold change compared to wild type NL4-3				
	NL4-3	A128T	Y99H	H171Q	T174I	N222K
EVG	2.2 ± 1.2	1	1	1	1	1
BI-224436	51 ± 17	6	2	4	706	2
S-I-82	12 ± 3	4	2	2	15	1
BDM-2	8.7 ± 2.8	4	4	3	253	2
MUT871	3.1 ± 1.0	2	4	6	323	3
MUT872	45 ± 12	7	2	1	15	2
MUT884	15 ± 5	5	2	1	12	3
MUT916	20 ± 9	4	3	1	14	3

a Antiviral activity estimated by EC_50_ fold change of each mutant compared to the wild type NL4-3 HIV-1 clone. Fold-change values >10 are highlighted in dark gray and those between 5 and 10 in light gray. One INSTI (EVG) and two INLAIs (BI-224436, S-I-82) were used as references.

We also analyzed the impact of the INLAI resistance mutations on viral fitness. The most detrimental mutation for INLAI activity, T174I, also had the greatest negative impact on replication fitness, reducing it to ~29% of that of wild-type control (Fig. S1). All remaining mutations, Y99H, N222K, A128T, and H171Q, that resulted in moderate INLAI resistance had moderate impact on replication fitness (Fig. S1).

### No antagonism between BDM-2 and antiretroviral drugs currently used in clinic.

**FIG 3:**
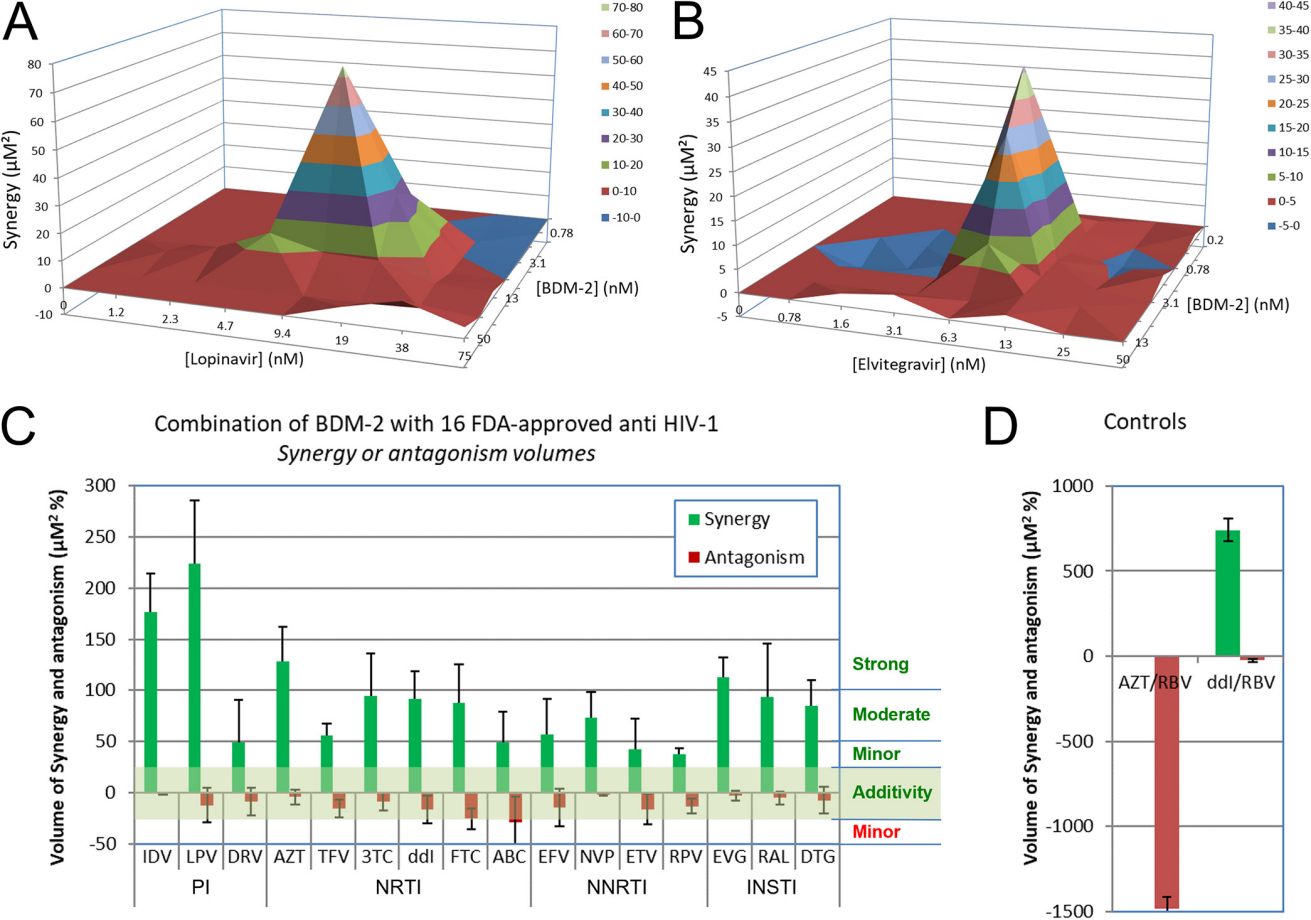
MacSynergy plot analyses to detect interactions between BDM-2 and 16 approved antiretroviral drugs. (A and B) MacSynergy plots of antiviral activity obtained after treatment of NL4-3-infected MT4 cells by combination of drugs, (A) with BDM-2 plus lopinavir, and (B) with BDM-2 plus EVG. (C) Quantitative results of synergy or antagonism volume by combination of BDM-2 with 16 clinical antiretroviral drugs of different classes. (D) Combinations of didanosine (ddI) + ribavirin (RBV) and azidothymidine (AZT) + RBV used as controls of synergy and antagonism, respectively.

All efficient treatments against HIV-1 infection are combination therapies of several (typically three for the highly active antiretroviral therapy, HAART) antiretroviral drugs with orthogonal modes of action. Therefore, it was important to confirm that BDM-2, as the lead compound of the new series of INLAIs, did not antagonize other antiretroviral drugs. To this end, we evaluated the activity of BDM-2 in the presence of approved antiretroviral drugs against NL4-3 HIV-1 clone replicating on MT4 cells. Examples of MacSynergy plots obtained for the combinations of BDM-2 with the protease inhibitor lopinavir (LPV) or the INSTI EVG are shown in Fig. 3A and B, respectively. The results indicated no antagonism of ascending concentrations of BDM-2 with the antiviral activity of LPV or EVG and *vice versa*. Next, we extended these analyses to a panel of 16 FDA-approved antiretroviral drugs, including 3 protease inhibitors, 6 NRTIs, 4 NNRTIs, and 3 INSTIs. In contrast to the drug interaction control (ribavirin + azidothymidine; Fig. 3D), BDM-2 displayed no antagonism with any of the 16 drugs. Moreover, BDM-2 displayed strong synergy with LPV and moderate synergy for some of the remaining drugs, including the INSTIs (Fig. 3C).

### Cocrystal structures of the BDM-2 series with HIV-1 IN.

**FIG 4:**
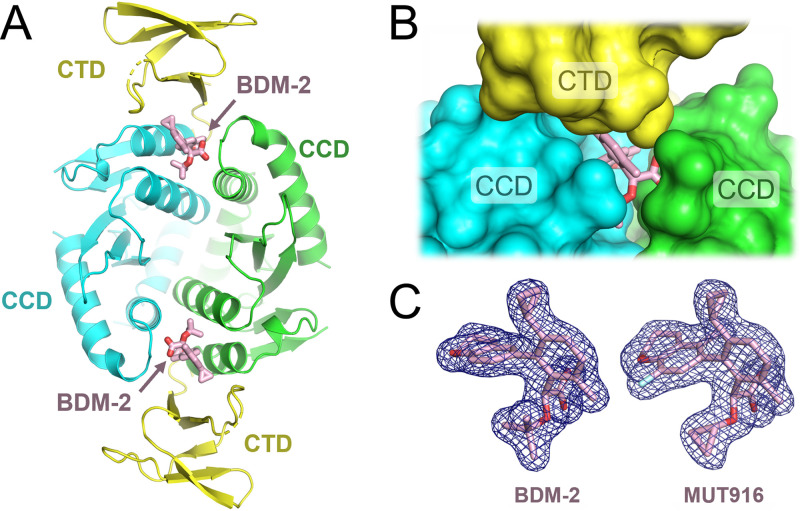
Two-domain CTD-CCD HIV-1 IN construct cocrystallized with BDM-2. (A) Overall structure of the BDM-2 containing complex comprising IN CCD dimer with two associated with CTDs. Each CCD-CTD interface sandwiches an INLAI molecule. The protein chains are shown as cartoons with CCD chains in cyan and green, and CTD in yellow. On all panels, INLAI molecules are shown as sticks with carbon, oxygen, and fluorine atoms in pink, red, and light cyan, respectively. (B) A view on one of the INLAI-induced CTD-CCD interfaces with protein chains shown in space-fill mode. (C) The final weighted *2Fo-Fc* electron density of BDM-2 (left) and MUT916 (right) contoured at 1σ and shown as blue mesh.

**FIG 5:**
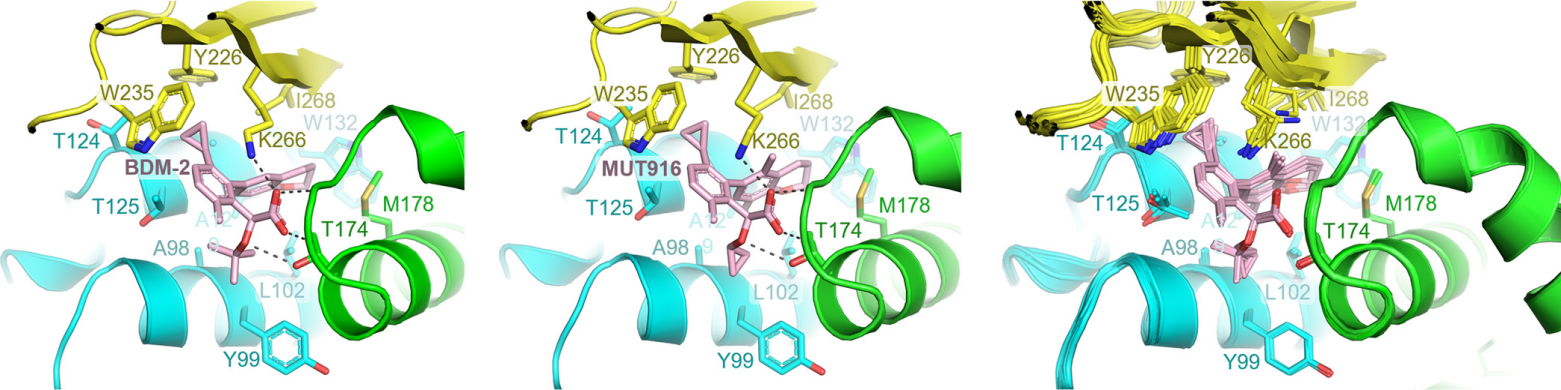
Details of the HIV-1 IN CTD-CCD interface induced by the BDM-2 series INLAIs. Left two panels show cocrystal structures containing BDM-2 and MUT916. The rightmost panel shows superposition of the 10 crystallographically independent CCD-CTD interfaces observed in five cocrystal structures with the two-domain HIV-1 IN construct. Selected residues are shown as sticks and indicated.

Acting as molecular glues, INLAIs induce a pathological interface involving HIV-1 IN CCD dimer and the CTD leading to hyper-multimerization and aggregation of the viral protein (39–42). To visualize the details of the interactions between the BDM-2 series of compounds and HIV-1 IN, we took advantage of a two-domain construct spanning the CTD and the CCD of HIV-1 IN, recently developed for X-ray cocrystallography with INLAIs (45). We crystallized this protein construct in the presence of the small molecules and refined the resulting cocrystal structures containing BDM-2, MUT871, MUT872, MUT884, and MUT916 to 1.8, 1.7, 2.1, 2.4, and 1.8 Å resolution, respectively (Table S1; Fig. 4 and 5). In addition, we refined a cocrystal structure of isolated HIV-1 IN CCD dimer with our lead compound BDM-2 to 2.0 Å resolution (Table S1; Fig. S2). While the latter structure clarifies the interactions made by BDM-2 within the principal INLAI-binding pocket on the HIV-1 IN CCD dimerization interface, the cocrystal structures with the two-domain construct additionally visualize the key INLAI-induced CTD-CCD contacts. Similar to what was observed with BI-D and STP0404 (45), the asymmetric units of the two-domain cocrystal structures each contained a single CCD dimer with a pair of associated CTDs. Every CCD-CTD interface buried a molecule of INLAIs, well-defined in an electron density map (Fig. 4C).

As shown for all other INLAIs (10, 12, 14, 21, 31), the small molecules engage the well-characterized pocket on the CCD dimerization interface, also implicated in the interaction with LEDGF/p75 IBD (38). The contacts made by all compounds within the CCD pocket are very similar, regardless of the IN construct used (Fig. S3). The INLAIs are anchored through hydrogen bonds with main chain amides of Glu170 and His171, and additionally with Thr170 side chain (Fig. 5, Fig. S2 and S3). In addition, the INLAIs engage in extensive hydrophobic interactions with IN CCD residues Leu102, Thr124, Thr125, Ala128, Ala129, Trp132, Ala169, Gln168, and Met178 (Fig. 5, Fig. S2 and S3). In particular, the *tert*-butoxy groups of BDM-2 and MUT871 make van der Waals contacts with Thr174, explaining why a substitution to a bulkier Ile residue at this position causes high-level resistance to these compounds (Table 9). In contrast, in MUT872, MUT884, and MUT916, the *tert*-butoxy group is replaced by a more compact cyclopropyloxy group (Fig. 5), which may allow sufficient space to accommodate the Ile side chain, explaining higher activity of these three compounds against T174I HIV-1 variants compared to BDM-2 and MUT871 (Table 9).

The molecules protrude from the HIV-1 IN CCD cleft to function as molecular glue for the recruiting of the CTD (Fig. 4A and B and Fig. 5). As recently shown for BI-D, STP0404, and BI-224436 (42, 45), BDM-2 and the analogs make hydrophobic contacts with the side chains of the CTD residues Trp235, Tyr226, Lys266, and Ile268. The carboxylate groups of the INLAIs form salt bridges with the Lys266 side chain (Fig. 4). However, the interactions of the BDM-2 INLAIs with Tyr226 are mediated by cyclopropyl sidechains, and therefore lack the aromatic stacking component that was prominent in the case of BI-D, which features a bicyclic aromatic scaffold (Fig. 1) (45). Moreover, the compact nature of the benzene scaffold and small size of the cyclopropyl side chain allow the side chain of Trp235 to approach closer to the CCD than in complexes stabilized by STP0404 and BI-D, resulting in a shift of Trp235 Cα atom by 1.4 and 1.9 Å, respectively, concluding in a pronounced pivoting of the entire CTD domain (Fig. S4, Movie S1).

## DISCUSSION

In this report, we described biochemical and antiviral properties of BDM-2 and its analogs MUT871, MUT872, MUT884, and MUT916 that collectively comprise a new series of INLAIs. Our results show that these small molecules possess strong anti-HIV-1 activity in T-lymphoblastoid cell lines as well as in primary human T-lymphocytes. Encouragingly, the potency of the BDM-2 series INLAIs is comparable to that of second-generation INSTIs with very high selectivity ratios, demonstrating negligible cytotoxicity (Table 2). Importantly, the compounds retained full activity against a panel of HIV-1 variants resistant to currently used antiretroviral drugs (Table 7). We observed no antagonism between the BDM-2 series INLAIs and the clinically approved antiretroviral drugs and detected synergy with the protease inhibitor lopinavir and INSTIs (Fig. 3).

INLAIs can be affected by naturally occurring amino acid sequence polymorphisms between circulating HIV-1 strains. While the interaction with the CTD involved highly conserved and invariant HIV-1 IN residues (Fig. 5) (45), the lining of the CCD pocket is subject to naturally occurring variability, which can affect susceptibility to INLAIs. In particular, the widespread polymorphisms at HIV-1 IN amino acid positions 124 and 125 can be problematic for INLAIs. Thus, S-I-82 was severely compromised in its ability to inhibit HIV-1 *NA* variants (Table 5 and 6). Likewise, we previously showed that HIV-1 strains with an Ala residue at IN position 125 were much less susceptible to MUT-A (16). In contrast, BDM-2 and its analogs maintained antiviral activity against an extended panel of 124/125 polymorphic strains (Table 7). Although INLAIs, including the BDM-2 series, form weak hydrophobic interactions with the HIV-1 IN residues 124 and 125 (Fig. 5, Fig. S2C and S3), these contacts are unlikely to be critical for binding to the CCD dimer. Indeed, the 124/125 polymorphisms did not affect the ability of MUT-A to inhibit IN-LEDGF/p75 interaction, which requires strong binding to the CCD pocket (16). Instead, the mutations reduced hyper-multimerization of IN in the presence of MUT-A. Our cocrystal structures with the two-domain HIV-1 IN construct revealed considerable pivoting of the CTD with respect to the CCD dimer depending on the small molecule mediating the interface (Fig. S4, Movie S1). Thus, for some INLAIs, changes at the 124/125 positions may be detrimental to stability of the CTD-CCD interface. It would be of interest to study the details of the CTD-CCD interfaces mediated by INLAIs that are sensitive to 124/125 polymorphisms.

Our results highlight that the ability to inhibit the IN-LEDGF/p75 interaction does not fully correlate with their potency as HIV-1 inhibitors (Tables 1, 2, and 4). We note that such dichotomy can be expected for molecular glues. Indeed, while the former property requires only strong binding of the compound to the IN CCD pocket, the latter depends on formation of a quaternary complex and fitness of the molecular surfaces. The INLAI cocrystal structures with two-domain HIV-1 IN constructs reported here (Fig. 4 and 5) and elsewhere (42, 45) will inform further development of INLAIs with improved antiviral properties. Although INLAIs predominantly function as maturation inhibitors, their ability to inhibit formation of the IN-LEDGF/p75 complex may lead to novel therapeutic approaches. LEDGF/p75 is the main cellular cofactor of IN that targets HIV-1 integration in actively transcribed genes (1). Debyser and colleagues proposed that inhibitors of the IN-LEDGF/p75 interaction may be useful in a block-and-lock strategy by retargeting HIV-1 integration to sites that are less susceptible to reactivation (29, 49). INLAIs which are particularly potent at inhibiting IN-LEDGF/p75 interactions, such as MUT871 and BDM-2 could be considered potential candidates for this approach. Indeed, MUT871 dissociated the IN-LEDGF/p75 complex with an IC_50_ of 14 nM (Table 1) and displayed a comparatively high antiviral activity in the single-round assay, with an EC_50_ of 630 nM (Table 4). Our HIV-1 IN-INLAI cocrystal reported here (Fig. 4 and 5) and elsewhere (45) may inform the design of research tools to inhibit the IN-LEDGF/p75 interaction without promoting IN aggregation. Interestingly, most of the BDM-2 series of INLAIs appeared to inhibit the interaction between full-length HIV-1 IN and LEDGF/p75 with higher potency than the interaction between the respective minimal binding domains (Table 1). In contrast to full-length HIV-1 IN, which forms stable tetramers (24, 43, 44), isolated CCD is only known to dimerize (50). We speculate that tetramerization of full-length IN may be slightly beneficial for the binding of BDM-2 and some of its analogs. Indeed, KF116, a pyridine-based INLAI, was reported to selectively target HIV-1 IN tetramers (51).

Most BDM-2 resistance mutations selected by the dose-escalation method mapped to the principal INLAI binding pocket on the CCD dimer (Table 9, Fig. 5, and Fig. S2). Explained by the interactions within the pocket, these mutations affect all INLAIs to various extents (10, 23, 33). Of these, T174I is the most detrimental, leading to a substantial loss in the antiviral activity of BDM-2 (Table 9). Thr174 makes hydrogen bonding and van der Waals interactions with the *tert*-butoxy moiety present in BDM-2 and shared by all potent INLAIs reported to date (Fig. 5, Fig. S2 and S3). A larger Ile side chain in position 174 is expected to clash with the *tert*-butoxy moiety explaining the loss of compound binding. Therefore, it is very encouraging that substitution of the *tert*-butoxy group for a more compact cyclopropyloxy in MUT872, MUT884, and MUT916, reduced the level of resistance associated with T174I mutation (Table 9). These results indicate that the CCD binding functionality of INLAIs may benefit from further development.

The surface of the CTD participating in the INLAI-induced interface with the CCD dimer is involved in multiple functions during the lentiviral integration process, explaining its high level of conservation (45). Strikingly, the triad of the CTD residues directly recruited by INLAIs (Tyr226, Trp235, and Lys266) are conserved in most lentiviruses. Therefore, it is not surprising that INLAI resistance mutations in the CTD have not been described. Our selection experiment revealed a single point mutation outside the CCD, N222K, which enabled a low level of resistance to BDM-2 and its analogs, as well as to BI-224436 (Table 9). Likewise, N222K was previously reported for its association with mild resistance to INLAIs (23, 39). Positioned ~14 Å from the bound inhibitor in our two-domain cocrystal structures, Asn222 is not involved in the INLAI-induced CTD-CCD interface. More research is required to explain if N222K has an allosteric affect indirectly reducing CTD surface complementarity with the CCD dimer, or possibly by reducing interactions of IN protomers as part of the hinge region between the α-helical CCD-CTD linker and the CTD.

To date, there is still no INLAI in advanced clinical investigation for proof-of-concept studies in patients infected with HIV-1 in phase IIa and phase III clinical trials. BI-224436 from Boehringer Ingelheim originally was introduced in phase I clinical trial, but this trial was interrupted for an unknown reason (52). Gilead reported in 2017 a very active INLAI compound with a benzothiazole core group, GS-9822 (47); however, this compound provoked renal and urinary bladder toxicity, which precluded further clinical investigation (47). VIIV also developed a new series of very active INLAIs, tetrahydronaphtyridines (53); however, these compounds have not yet reached clinical trials. Recently, ST Pharm (Seoul, South Korea) reported STP0404 (Pirmitegravir), a very promising INLAI (21), which has now completed clinical phase I study (22). Well tolerated and with favorable pharmacokinetics suitable for once-daily low dose regimen, the compound is expected to move onto a phase IIa trial.

After completion of preclinical studies (data not shown), BDM-2 has been further investigated in a single ascending dose phase I clinical trial investigating safety, tolerability, and pharmacokinetics in healthy male subjects completed in 2020. The results of this trial were reported on 17 June 2020 without any serious adverse event and few mild adverse events (reference 20 and manuscript in preparation). Therefore, BDM-2 is together with Pirmitegravir the most advanced INLAI in investigation today in humans, which supports further clinical investigation.

## MATERIALS AND METHODS

### Compound synthesis.

BDM-2, MUT871, MUT872, MUT884, and MUT916 were synthesized at Biodim (Romainville, France) as described in patent application WO2015/001125A1, according to examples 13, 38, 24, 40 and 44, respectively. The reference compound BI-224436 was prepared as described in patent application WO2009/062285A1, according to compound 1144; S-I-82 was prepared as described in WO2013/062028A1 according to compound I-82.

### Reference compounds.

Control compounds such as nevirapine (NVP), efavirenz (EFV), and azidothymidine (AZT) were obtained from the NIH AIDS Research and Reference Reagent Program. Raltegravir (RAL), dolutegravir (DTG), nevirapine (NVP), indinavir (IDV), AZT, ribavirin (RBV), lopinavir (LPV), darunavir (DRV), tenofovir (TFV), lamivudine (3TC), didanosine (ddI), emtricitabine (FTC), abacavir (ABC), efavirenz (EFV), etravirine (ETV), rilpivirine (RPV), and elvitegravir (EVG) were purchased from Selleck Chemicals.

### Molecular biology and biochemistry.

Epitope-tagged proteins used in IN-LEDGF/p75, CCD-IBD interaction and IN multimerization assays were constructed and purified as described previously (12). HTRF-based CCD-IBD interaction, IN-LEDGF/p75 interaction and IN multimerization assays were performed as described previously (12).

### Cell culture.

MT4 cells were obtained through the AIDS Research and Reference Reagent Program, Division of AIDS, NIAID, NIH. MT4 cells were grown in RPMI 1640 supplemented with 10% heat-inactivated fetal calf serum and 100 IU/mL penicillin, and 100 μg/mL streptomycin (Invitrogen) to obtain RPMI-complete medium.

### Virus strains and recombinant HIV-1 molecular clones.

HIV-1 NL4-3 and NL4-3Δ*env*-luc molecular clones were obtained from the NIH AIDS Research and Reference Reagent Program.

### Viral stocks.

Viruses were prepared and quantified as described previously (12) in 293T cells; single-round viral stocks were produced by cotransfecting pNL4-3Δ*env* with VSV-G envelope expression vector (12). HIV-1 resistant mutants were constructed as described previously (16).

### Antiviral assay in MT4 cells (multiple round infection assay).

MT4 cells growing exponentially at the density of 10^6^/mL were infected with HIV-1 strain NL4-3 at a multiplicity of infection (MOI) of 0.001 for 2 h in the presence of different concentrations of compounds, and the CellTiter-Glo luminescent reagent (Promega) was used to quantify cell viability as described previously (12). To evaluate the effect of the human serum on the antiviral potency on INLAIs and other compounds used as references, we measured their EC_50_ and EC_90_ from the dose response curve in the presence of various concentrations of human serum between 0% to 50% and linearly extrapolated their value at 100% human serum for PA-EC_90_ determination.

### Replication-defective HIV assay (single round infection assay).

MT4 cells (growing exponentially at the density of 10^6^/mL) were infected with VSV-G-pseudotyped NL4-3Δ*env*-luc at an MOI of 0.0001, and Luciferase expression was quantified after 2 days using the One-Glo luciferase assay (Promega), as described previously (12).

### Cytotoxicity assays.

Growth inhibition was monitored in a proliferating human T-cell line (MT4) with different concentrations of compounds, using the CellTiter-Glo luminescent reagent (Promega), as previously described (12).

### Resistant virus selection.

MT4 cells infected with HIV NL4-3 isolate were cultured in the presence of BDM-2 at the EC_50_ concentration determined earlier. At each passage, cells from original culture in the presence of inhibitor were mixed with equal amount of no-drug control cells to propagate, and viral replication was monitored by the production of p24 antigen in the supernatant. Inhibitor concentration was gradually increased at each passage. At three different times, early (day 18), intermediate (day 42), and final passage (day 66 to 73), viral RNA was extracted using QIAexpress (Qiagen) and IN sequences were determined by RT-PCR as a bulk. The quantitative estimate of each mutation induced at each passage mentioned above was determined as a percentage of total mutations detected in the IN sequences as a bulk.

### Viral replication capacity.

HIV-1 recombinant viruses harboring various single point INLAI resistant mutations (A128T, Y99H, N222K, T174I, H171T, L102F, and T124D) in an NL4-3 background were used to infect MT4 cells with equivalent p24 quantity and compared with infection of MT4 cells in same conditions with identical p24 quantity of wild type NL4-3 and of some INSTI-resistant viruses (G140S and Q148H). Replication kinetics of the INLAI resistant variants were compared and viral production was determined by p24 assay daily for 5 days.

### Combination antiviral activity assays.

BDM-2, as lead compound representative of the series, was used in these experiments. Combination studies were performed in MT4 cells infected with NL4-3, as previously described (3). Multiple concentrations of BDM-2 were tested in checkerboard pattern in the presence and absence of dilutions of 16 representative approved anti-HIV drugs of different classes. Compound combinations were analyzed by calculations to quantify deviation from additivity at the 50% level. Data were analyzed as described by Prichard and Shipman by using the MacSynergy II program (54). Synergy volumes in the range of −25 to +25 define additivity; −50 minor antagonism, +50 minor synergy, and up to 100 and >100 moderate and strong synergy, respectively. Combinations of RBV with AZT or ddI were used as controls for antagonism and synergy, respectively.

### Cocrystallization of BDM-2 with isolated HIV-1 IN CCD.

Expression and purification of the HIV-1 IN CCD F185K (50-212) were performed as previously described (12) and used for crystallization. Briefly, the sequence of HIV IN CCD F185K (50-212) with a N-terminal HRV3C Prescission protease cleavage site was cloned in pGEX-6P by enzymatic restriction through BamHI and XhoI sites and overexpressed in Escherichia coli BL21(DE3) STAR cells. Next, 4 L of LB (100 mg/L ampicillin) were induced at an optical density at 600 nm(OD600) of 0.6 to 0.8 with 0.5 mM IPTG for 18 h at 18°C. Cells were harvested, resuspended in lysis buffer (50 mM HEPES pH 7.5, 0.5 M NaCl, 5 mM MgCl_2_, 5 mM DTT) at a ratio of 10 mL of buffer per gram of biomass, in the presence of 1 mM PMSF and Roche Complete inhibitor cocktail tablets to avoid protease degradation. Lysis was performed by pulse sonication every 2 s at 40% amplitude for 1 min/g of cells at 4°C. The lysate was then clarified by ultracentrifugation for 1 h at 125,000 *g* at 4°C. After filtration through a 5-μm cellulose filter, the sample was loaded on a 5-mL GSTrap FF column (Cytiva). The column was washed with lysis buffer, and proteins were eluted by on-column cleavage of the GST tag using 2 mg HRV14 3C protease for 18 h at 4°C. The protein was then concentrated on Amicon (10-kDa MWCO) and further purified using a Superdex 75 10/300 GL column (Cytiva) equilibrated with GF buffer (50 mM MES pH 5.5, 50 mM NaCl, 5 mM DTT). Fractions containing the purest protein were pulled together and concentrated on Amicon (10 kDa MWCO) and used for crystallization.

Crystals were grown at 20°C by vapor diffusion using 3 μL of protein at 5 mg/mL in 50 mM MES pH 5.5, 50 mM NaCl, 5 mM DTT mixed to 3 μL of reservoir solution containing 0.1 M sodium cacodylate pH 6.5, 1.26 M ammonium sulfate, with 500 μL of reservoir solution in the well (16). Crystals grew within a week and were soaked by adding 0.1 μL of 12 mM ligand/BDM-2 at 20°C to the drop 8 h. Crystals were cryo-protected in oil (FOMBLIN Y LVAC 14/6; Aldrich) for a few seconds and cryo-cooled in a stream of liquid nitrogen at 100 K. Data collection was conducted using an in-house Rigaku FR-X diffractometer equipped with a rotating anode and a Dectris Eiger R 4M detector. X-ray diffraction images were indexed and scaled with XDS (55). Structure solving and refinement were performed using PHENIX program suite (56).

The structure was solved by molecular replacement using Phaser (57). It was refined with phenix.refine (58). The ligand BDM2 coordinates and restrains were generated with Elbow (59) and placed in the structure using LigandFit (60). Structure superpositions were performed in Coot (61). All structure drawings were performed with PyMOL (62) and Coot (61). A 2D view of ligand interactions has been generated with LigPlot (63). Statistics of data scaling and structure refinement are summarized in Table S1; an example of final electron 2*Fo-Fc* density map is shown in Fig. S2B.

### X-ray crystallography of the INLAIs with a two-domain (CTD-CCD) HIV-1 IN construct.

IN-INLAI complexes were prepared and crystallized as described in Singer et al. (45). The protein construct spans HIV-1 IN CTD (residues 220 to 288) with the W243E monomerizing mutation, and CCD (residues 50 to 212) with the solubilizing F185K mutation. Here, the C-terminal tail of the CTD (residues 270 to 288) acts as a flexible linker joining the domains. The CTD-CCD protein was expressed with an N-terminal hexa-histidine-Sumo tag in E. coli BL21-CodonPlus (DE3) cells (Agilent). The protein was purified by affinity chromatography on Ni-NTA Sepharose (Qiagen), followed by cation exchange chromatography using the HiTrap SP HP column (Cytiva) and size exclusion chromatography through a HiLoad 16/600 Superdex 200 pg column (Cytiva) with the elution buffer containing 0.5 M NaCl and 20 mM HEPES-NaOH pH 7.5.

Stock solutions of BDM-2, MUT871, MUT872, MUT884, and MUT916 for cocrystallization experiments were prepared in dimethyl sulfoxide at concentrations of 40 mM. W243E/F185K CTD-CCD (0.6 mg/mL) was supplemented with 25 μM INLAI and concentrated to 5 mg/mL using a 10-kDa cutoff VivaSpin device (Sigma-Aldrich). Crystals were grown at 18°C in sitting or hanging drops formed by combining 1 μL protein with 1 μL reservoir solution, which contained 3.75 to 6% (wt/vol) polyethylene glycol 8,000, 12 to 25% (wt/vol) ethylene glycol, 30 mM MgCl_2_, 30 mM CaCl_2_, and 0.1 M imidazole 2-(N-morpholino)ethanesulfonic acid, pH 6.5. Crystals, cryoprotected in mother liquor supplemented with 30% ethylene glycol, were flash-frozen by plunging into liquid nitrogen.

Diffraction data were collected at beamline I04 of the Diamond Light Source (Oxford, UK) at 100 K, using a wavelength 0.9795 Å, 100% transmission, a 43 × 30-μm beam, with 0.1-s exposure and 0.15 to 0.3° rotation per image. DIALS (64) within the Xia2 data processing pipeline (65) was used for integration, scaling, and merging of the diffraction data. Structures were solved by molecular replacement in PHASER (57) within the Phenix software suite (56) using HIV-1 IN CTD-CCD structure from PDB entry 8A1P (45) as a search model. Multiple rounds of interactive fitting in Coot (61) and refinement in phenix.refine (version 1.20.1-4487-000) (58) allowed adjustment of the resulting models. Initially protein chains were extended, followed by the addition of water, polyethylene glycol and ethylene glycol molecules visualized in the electron density map. INLAI molecules were then fitted into prominent positive difference densities. eLBOW (59) and PRODRG (66) were used to generate ligand geometry definition files. The TLSMD server (67) guided the generation of translation/libration/screw (TLS) anisotropic B factor groups for the protein chains. MolProbity (68) was used for assessment of the refined models, which fit well into the electron density and have good geometry (Table S1, Fig. 4C).

### Data availability.

The refined co-crystal structures along with associated diffraction data were deposited with the protein databank under accession codes 8BV2, 8CBR, 8CBS, 8CBT, 8CBU, and 8CBV.
