# Supplementary material for: Biological and Structural Analyses of New Potent Allosteric Inhibitors of HIV-1 Integrase
Source: Antimicrob Agents Chemother. 2023 Jun 13;67(7):e00462-23. doi: 10.1128/aac.00462-23 (PMC10353390; doi:10.1128/aac.00462-23)
Supplement: Supplemental file 1 — Supplemental material. Download aac.00462-23-s0002.docx, DOCX file, 1.9 MB [file aac.00462-23-s0002.docx]

**Biological and structural analyses of new potent allosteric inhibitors of HIV-1 integrase**

Damien Bonnard^1,°,¶^, Erwann Le Rouzic^1,°,¶^, Matthew R Singer^2,¶^, Zhe Yu^2,#^, Frédéric Le Strat^1°^, Claire Batisse^3^, Julien Batisse^3^, Céline Amadori^1,4^, Sophie Chasset^1°^, Valerie E. Pye^2^, Stéphane Emiliani^4^, Benoit Ledoussal^1,&^, Marc Ruff^3^, François Moreau^1°^, Peter Cherepanov^2,5,§^, and Richard Benarous^1,§,*^

# Supporting information


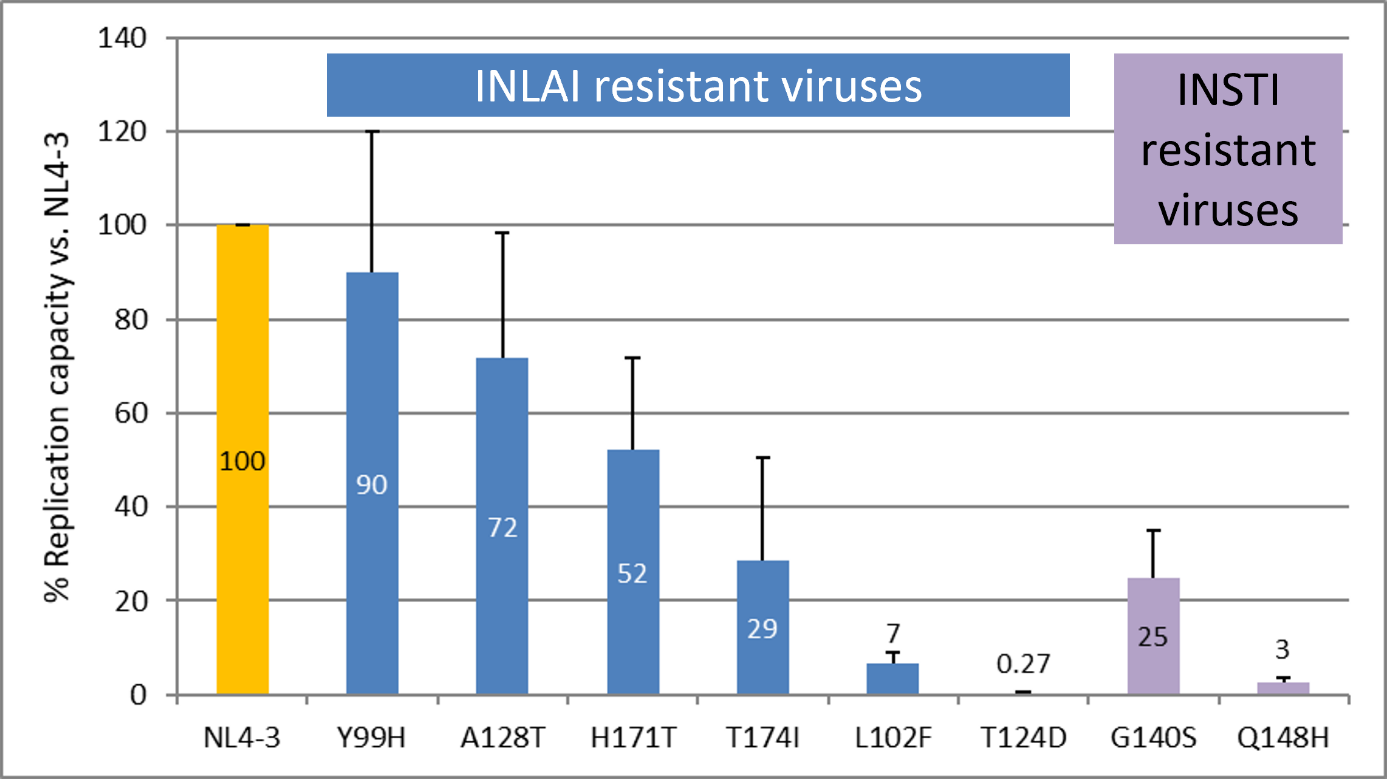


**FIGURE S1** Replication capacity of INLAI-resistant viruses. Replication capacity of HIV-1 NL4-3 carrying resistance mutations to INLAIs tested by their infectivity on MT4 cells. Infectivity was standardized by the quantity of p24 antigen from each virus. Comparison is shown with NL4-3 wild type and NL4-3-INSTI resistant viruses, G140S, Q148H, used as controls. Replication capacity is estimated as a percentage of wild type NL4-3 infectivity (in orange). Data are mean ± SD of >3 independent experiments.

^
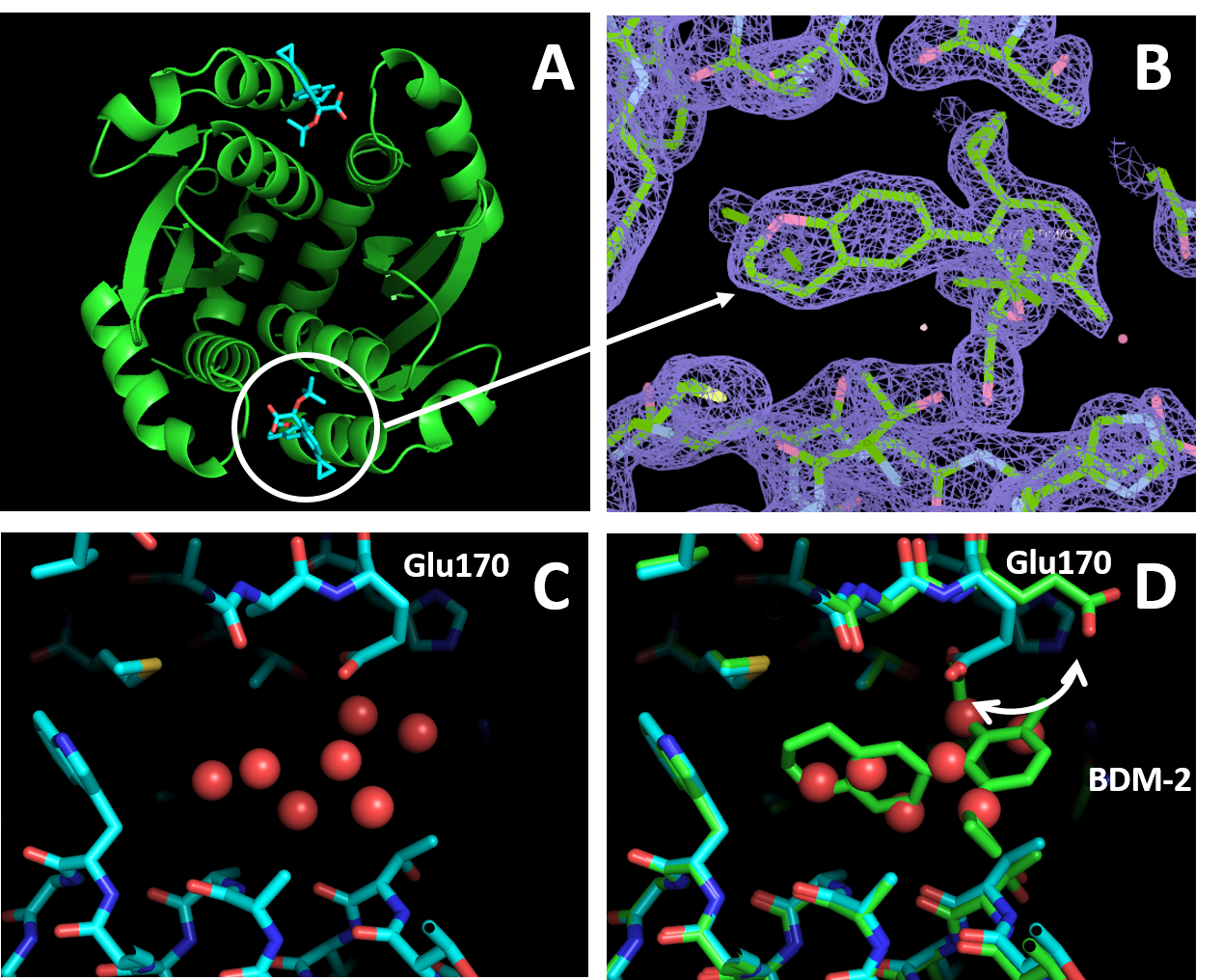
^

**FIGURE S2** BDM-2 co-crystallized with isolated CCD. (A) full view of the IN CCD dimer, main chain in green and BDM-2 in blue. (B) Enlargement of the BDM2 binding pocket with the electronic density. (C) Close view of the structure of the IN-CCD (in blue) LEDGF binding pocket without ligand (PDB 4LH4). The pocket is filled with water molecules (red). (D) Superposition of the ligand binding pocket without (blue) and with (green) BDM-2. The arrow shows the movement of Glu170 side chain upon BDM-2 binding.

^
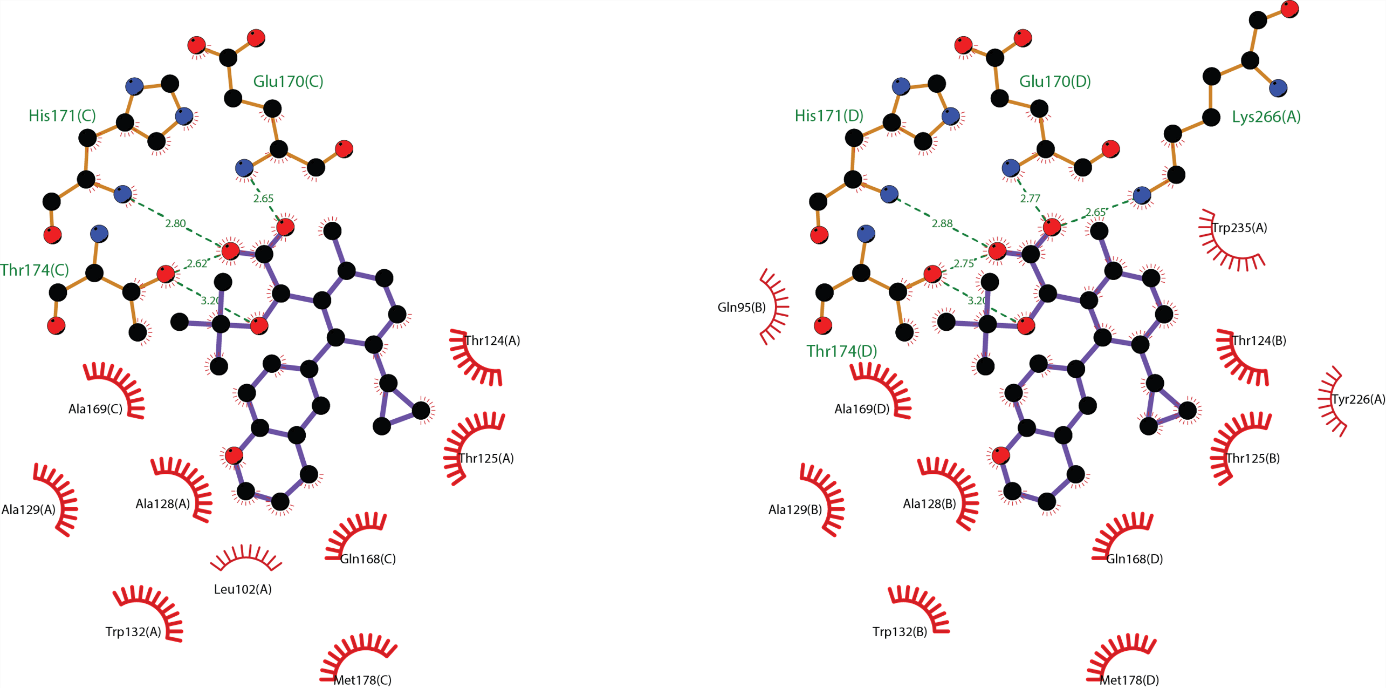
^

**FIGURE S3** Ligplot analysis of BDM-2 interactions with IN in co-crystal structures with isolated CCD (left) and CTD-CCD two-domain construct (right).


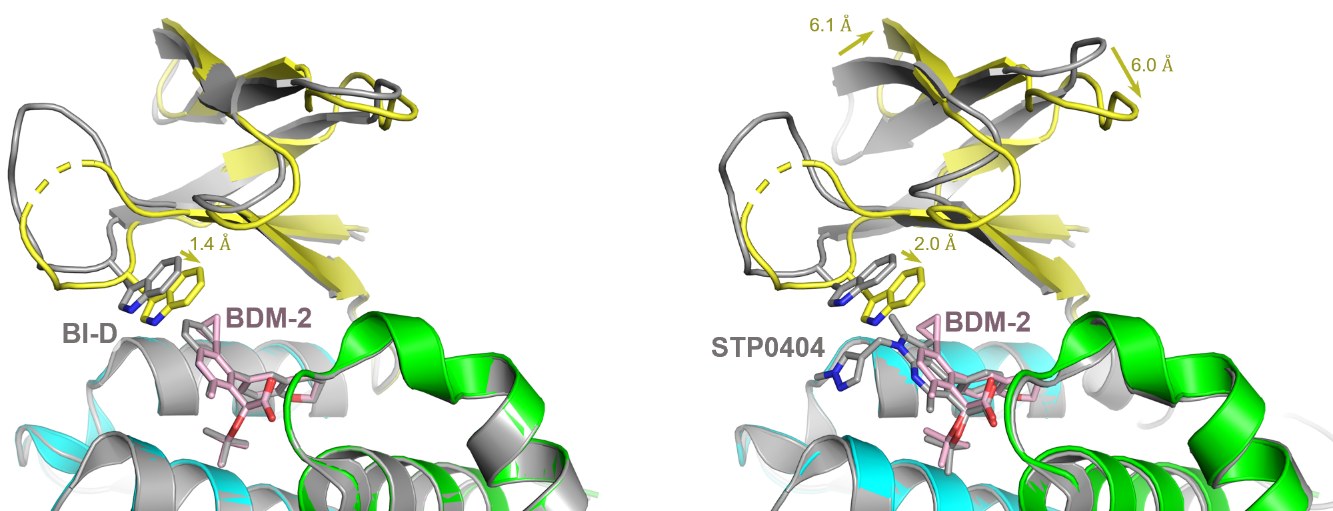


**FIGURE S4** Conformational adaptation of the CTD-CCD construct in co-crystals with different INLAIs. Superposition of the CCD portion of BDM-2 co-crystal structure with BI-D (left) and STP0404 (right) co-crystal structures. Protein chains are depicted in cartoon representation, and small molecules and Trp235 are shown in sticks and indicated. In both panels, the BI-D co-crystal structure is shown in color and style as in Fig. 4A. Cartoons and carbon atoms on the BI-D and STP0404 co-crystal structures are grey. Note the displacement of the Trp325 side chain. Local displacements of the CTD domain are indicated with arrowheads. See also Movie S1.

**MOVIE S1** Displacement of the CTD domain between co-crystal structures of the CTD-CCD construct with STP0404 and BDM-2 (.mov)

**TABLE S1** Crystallographic X-ray data processing and refinement statistics

|  | **BDM-2/CTD-CCD** | **MUT871/CTD-CCD** | **MUT872/CTD-CCD** | **MUT884/CTD-CCD** | **MUT916/CTD-CCD** | **BDM-2/CCD** |
| --- | --- | --- | --- | --- | --- | --- |
| **Data collection:** |  |  |  |  |  |  |
| **Wavelength (Å)** | 0.979499 | 0.979499 | 0.979499 | 0.979499 | 0.979499 | 1.542 |
| **Space group** | P21 | P21 | P21 | P21 | P21 | P 31 2 1 |
| **Unit cell parameters** | | | | | | |
| ***a, b, c* (Å)** | 53.22, 65.17, 70.47 | 53.23, 64.85, 70.44 | 53.31, 64.47, 70.70 | 52.85, 63.56, 69.60 | 53.28, 65.08, 70.59 | 72.57, 72.57, 65.965 |
| **α, β, γ (^o^)** | 90, 101.48, 90 | 90, 101.50, 90 | 90, 101.66, 90 | 90, 100.72, 90 | 90, 101.64, 90 | 90, 90, 120 |
| **Number of crystals used** | 1 | 1 | 1 | 1 | 5 | 1 |
| **Resolution (Å)** | 52.16-1.80 (1.83-1.80) | 52.16-1.70 (1.73-1.70) | 64.47-2.14 (2.18-2.14) | 51.92-2.44 (2.48-2.44) | 65.08-1.82 (1.85-1.82) | 19.97 - 2.0 (2.072 - 2.0) |
| **Number of reflections** | | | | | | |
| **measured** | 2,028,572 (88,254) | 1,042,809 (50,936) | 962,204 (26,052) | 211,844 (9,599) | 1,970,532 (91,881) | 131,582 (13,489) |
| **unique** | 43,935 (2,208) | 51,832 (2,574) | 26,052 (1,264) | 17,040 (852) | 442,542 (2,137) | 13,873 (1,364) |
| **Completeness (%)** | 100 (100) | 100 (100) | 100 (100) | 99.98 (100) | 100 (100) | 99,19 (100) |
| **Multiplicity** | 46.17 (39.97) | 20.12 (19.79) | 36.95 (27.51) | 12.43 (11.27) | 46.32 (43.00) | 9.5 (9.9) |
| **<*I*/s(*I*)>** | 19.3 (0.4) | 26.1 (0.5) | 13.6 (0.7) | 17.9 (1.5) | 19.9 (0.6) | 57.43 (11.20) |
| ***R*_merge_ (%)** | 0.139 (1.933) | 0.050 (1.057) | 0.167 (1.720) | 0.084 (0.260) | 0.151 (1.974) | 0.02859 (0.1985) |
| ***R_pim_* (%)** | 0.021 (0.308) | 0.012 (0.242) | 0.027 (0.320) | 0.025 (0.077) | 0.023 (0.303) | 0.009974 (0.06627) |
| **CC ½** | 0.999 (0.906) | 1.000 (0.905) | 0.998 (0.931) | 0.999, 0.992) | 0.999 (0.939) | 1 (0.986) |
| **Refinement statistics:** | | | | | | |
| **Resolution range (Å)** | 52.16-1.80 (1.83-1.80) | 52.16-1.70 (1.73-1.70) | 52.21-2.14 (2.23-2.14) | 51.92-2.44 (2.59-2.44) | 38.11-1.82 (1.86-1.82) | 19.97 - 2.0 (2.072 - 2.0) |
| **Number of reflections** | | | | | | |
| **Total** | 43,291 (2,201) | 51,787 (2,751) | 26,023 (2,728) | 16,995 (2669) | 42,476 (2,696) | 13,873 (1,364) |
| **Free** | 2,138 (123) | 2,573 (129) | 1,258 (148) | 838 (131) | 2,093 (150) | 1,386 (137) |
| **R_work_/R_free_** | 0.1771/0.2112 | 0.1667/0.1980 | 0.2073/0.2534 | 0.2018/0.2536 | 0.1865/0.2297 | 0.1955/0.2262 |
| **Number of atoms** | | | | | | |
| **Total** | 3397 | 3490 | 3292 | 3287 | 3427 | 1157 |
| **Protein** | 3003 | 3145 | 3027 | 3066 | 3124 | 1020 |
| **Ligands** | 117 | 119 | 107 | 99 | 110 | 110 |
| **Solvent** | 197 | 226 | 158 | 122 | 193 | 35 |
| **R.m.s. deviations from ideal** | | | | | | |
| **bond lengths (Å)** | 0.006 | 0.017 | 0.002 | 0.002 | 0.006 | 0.008 |
| **bond angles (^o^)** | 0.789 | 1.447 | 0.402 | 0.471 | 0.835 | 1.03 |
| **Average B-factor (Å^2^)** | 48.6 | 51.63 | 58.49 | 58.45 | 48.51 | 31.65 |
| **Clash Score *^b^*** | 4.36 | 7.74 | 4.91 | 5.21 | 4.31 | 3.85 |
| **Favored Rotamers *^b^*** | 98.47 | 96.43 | 97.83 | 98.46 | 97.59 | 100 |
| **Ramachandran plot (%) *^b^*** | | | | | | |
| **Favored** | 98.9 | 97.06 | 98.32 | 97.79 | 98.37 | 98.32 |
| **disallowed** | 0 | 0 | 0 | 0 | 0.27 | 0 |

*^a^* Values in parentheses correspond to the highest resolution bin.
*^b^* Analyzed using MolProbity (http://molprobity.biochem.duke.edu/).
